# What should we expect from Switzerland’s compulsory dental insurance reform?

**DOI:** 10.1186/s12913-018-3065-2

**Published:** 2018-04-10

**Authors:** Enrico di Bella, Ivo Krejci, Stefano Ardu, Lucia Leporatti, Marcello Montefiori

**Affiliations:** 1 Department of Economics and Business Studies, University of Genoa, Via Vivaldi 5, 16126 Genoa, Italy; 2 CUMD, Division of Cariology & Endodontology, University of Geneva, Geneva, Switzerland; 3 Department of Political Science, University of Genoa, Genoa, Italy

**Keywords:** Dental insurance, Dental health, Dental reform, Public health policy, Switzerland

## Abstract

**Background:**

A vast and heated debate is arising in Switzerland as a result of some recent citizens’ initiatives aimed at introducing compulsory dental health care insurance. The Grand Conseils of the Vaud, Geneva, and Neuchâtel cantons recently approved three public initiatives and their citizens are expected to vote on the proposal in 2018. The process of collecting signatures has begun in several other cantons and the discussion has now moved to a national level.

**Discussion:**

At present, there is no scientific research that can help policy-makers and citizens to understand the main economic implications of such reform. We attempt to fill this gap by analysing three critical issues: the level and determinants of unmet needs for dental care in Switzerland; the protection of vulnerable individuals; and the economic sustainability of reform.

**Results and short conclusions:**

The results show that income is not a unique determinant of barriers to access to dental care but rather, cultural and socio-demographic factors impact the perceived level of unmet dental care needs. The reform might only partially, if at all, improve the equity of the current system. In addition, the results show that the 1% wage-based contribution that the reform promoters suggest should finance the insurance is inadequate to provide full and free dental care to Swiss residents, but is merely sufficient to guarantee basic preventive care, whereas this could be provided by dental hygienists for less.

## Background

Disparities in oral health and in the access to dental care dominate the international literature on the topic. The financial burden of out-of-pocket dental care expenditure gained attention due to its affordability and weight in household budgets because dental care is typically not covered by insurance schemes or it is at a lower level. Several studies show that children [14, 26], individuals with special needs [33], elderly people [19, 41], people living in rural areas [39] and low-income individuals generally [45] are more affected by oral diseases, such as dental caries and periodontitis. In addition, racial and ethnic minorities tend to experience disparities in oral health status [18]. These disparities might be considered a consequence partially of being intrinsic to the socio-demographic group and partially of the barriers to dental care access. Disadvantaged socio-demographic groups (e.g. low-income individuals and ethnic minorities) are less likely to have private dental coverage [28, 34] while dental care is barely included in social programmes. In several countries, being part of the labour force is strongly predictive of having dental coverage [29]. For example, in the US, dental insurance is excluded from the Medicare programme and medical insurance is 2.5 times more common than dental insurance; elderly Americans are often excluded from coverage because their employer-based insurance coverage expires when they retire [20]. Consequently, middle-aged groups tend to record higher levels of coverage while elderly people out of the labour force tend to be excluded from coverage [30]. In addition, the impact of oral health on the probability of receiving dental care is controversial: according to Bhatti et al. [2], people with poor oral health are less likely to use dental services, and this is probably the reason for their poor oral health but, among those using dental services, people with poor oral health tend to visit dentists more often than those with good oral health. Listl et al. [23], using Survey of Health, Ageing and Retirement in Europe (SHARE) data for a selection of European countries, found that reasons for dental non-attendance are different in different countries. In most countries (e.g. Italy, Spain, Greece, and Germany), several people do not use dental care because they perceive it as unnecessary; in other countries, a high percentage of people declare they do not use dental services because they are not affordable.

Given these considerations, previous research identified the presence of insurance as a key factor associated with the ‘use’ and ‘non-use’ of dental care. In general, having insurance seems to increase the use of dental services significantly, as it reduces the perceived price of care [1, 12, 27, 28, 31, 35]. However, it is not clear if the relationship is causal or if greater need leads patients to obtain insurance [1, 22]. Kreider et al. [20] highlighted the relevance of the selection problem that emerges when analysing the impact of insurance on dental care use: indeed, seeking dental care and obtaining insurance might be driven by unobserved factors (e.g. aversion to risk and expectations of future needs), making the relationship spurious. For this reason, recent approaches [7, 20, 32] have tended to use econometric specifications that can deal with the selection bias problem and have still produced results consistent with the previous ones.

In addition, differences in the use pattern of dental care might be caused by the different characterization of ‘private’ (i.e. offered through employers or other organizations) and ‘public’ systems (i.e. state-run). In ‘private systems’, buying health insurance different from the plan offered by an employer usually costs more, as there is no cost sharing with the employer. On the other hand, public health insurance is a government insurance system that pays the healthcare provider for medical care. Manning et al. [25] proposed an analysis on the effect on dental care use of different insurance schemes (free or co-insured by out-of-pocket expenditure by families). The authors found that passing from a 25% coinsurance rate to free dental coverage increased the probability of using dental services from 53.6% to 68.7%. Consistent results have been obtained by Mueller and Monheit [31], who also claimed that the increase in demand consequent to extending insurance is particularly relevant for expensive dental services (e.g. bridges and crowns) but less evident for basic treatments (e.g. X-rays and cleaning) that are consumed regardless of insurance status. This means that insurance tends to change the mix of dental services consumed.

During the last 2 years, the Swiss dental care system has been placed at the centre of a heated debate concerning a reform proposal to introduce compulsory dental care insurance [13]. The proponents of reform suggest financing dental insurance through a 1% contribution to wages based on the model of the *Assurance Vieillesse et Survivants*, a compulsory insurance intended to support retired people and the pensions of widows and widowers. The contribution should be equally divided between the employer and employee, although each canton might decide to impose a different financing principle. In the current system, dental care is not included in the healthcare system, except for costs generated by serious and unavoidable diseases of the masticatory system or by another serious illness or its aftermath. According to the Swiss Federal Office of Statistics, in Switzerland, the total cost of dental care in 2014 amounted to 4.1 billion Swiss francs (CHF), equivalent, on average, to approximately 492 CHF per capita per year. Patients themselves directly pay 90% of all dental care costs while the remainder is covered by social and private insurance or other regimes [5]. In the European framework, Switzerland ranks first in the level of out-of-pocket expenditure for dental care [37]. According to Listl et al. [23], in Switzerland, 28.4% of non-users of dental services perceive dental care as too expensive; this percentage is the highest recorded among the countries analysed and Switzerland’s neighbouring countries record definitely lower percentages (e.g. Austria = 11.1%; Germany = 4.4%; Italy = 18.4%; and France = 18.5%). A recent study by Guessous et al. [19] applied to survey data in the Canton of Geneva found that the prevalence of forgoing dental care is highly dependent on income level.

Between 2014 and 2016, three popular initiatives for reform of the current system were proposed in three cantons (Neuchâtel, Vaud, and Geneva) and the *Grand Conseils* of these cantons validated the initiatives so that the votation to determine citizens’ views on the reform will presumably take place by 2018. In the meantime, several other cantons are preparing popular initiatives on this subject. Thus, the debate is moving to national level with the following two main positions at stake. On the one hand, the proponents of the reform maintain that it will bring redistributive effects that will benefit the vulnerable part of society and that it will promote equitable access to dental care [24]. In particular, the promoters of the reform claim that low-income individuals tend to be excluded from private dental care (e.g. [19]) and that the introduction of a mandatory insurance scheme might improve access to dental care for individuals currently experiencing economic barriers to access dental services. Moreover, according to the proponents, easier access to dental care would improve prevention and check-up visits, thereby reducing long-run problems. On the other hand, opponents of the proposal, led by the Swiss Dental Association (Société Suisse d’Odonto-Stomatologie, SSO), do not favour compulsory dental coverage because they do not consider it the proper response to the inequity in dental care access. Rather, they consider that it would inflate the bureaucracy of the system and have undesirable effects, such as a substantial decrease in dental care quality and excess demand. Furthermore, the SSO is concerned that the reform would release many patients from their responsibility for taking care of their teeth by means of adequate preventive oral hygiene.

This debate is a novelty in the Swiss context and international experiences about the introduction of compulsory dental insurance within the last 2 decades are rare. Some evidence on the impact of recent dental reforms have been proposed in Thailand [40], Israel [36], and Chile [8]; however, results are not consistent in terms of impact of improving coverage on dental care utilization and inequalities. Consequently, the actual impact of this reform is not clear or easy to predict. This debate is a rare phenomenon in which dental care policy is being discussed at all levels and is of widespread interest for policy-makers to find new solutions. This study attempts to assess the feasibility and impact of providing free and comprehensive dental benefit to the general population of Switzerland focusing in particular on two critical issues of the reform proposal:

1. Is the reform proposal justified by the presence of relevant barriers to access dental care in Switzerland?
2. Can the suggested scheme for financing dental insurance guarantee the economic sustainability of the reform?

The Swiss healthcare system is particularly complex owing to the sharing of decision-making powers among three different stakeholders: three levels of government (i.e. confederation, cantons, and municipalities), corporatist bodies (including insurance companies and healthcare providers), and Swiss citizens, who can pervasively influence health policy-making through veto and popular initiatives. The scheme of financing the Swiss healthcare has been found to be one of the most regressive within the OECD countries [3, 11, 44, 46, 47]. Consequently, the reform proposal can be justified by a reduction in the unfairness of the entire healthcare system by reforming the financing scheme and reducing the perceived cost of dental services for vulnerable individuals. At the moment, four forms of financing co-exist (for a more in-depth description, see [9, 11]): Mandatory Health Insurance (MHI), government subsidies and benefits, General Social Insurance, and household expenditure. Since 1996, the MHI has been in existence to cover a comprehensive basket of healthcare services fixed at the federal level. The MHI services included in the benefits package have to be simple, economical, and appropriate (Loi fédérale sur l’assurance-maladie, LAMal). Households pay monthly premiums for private insurance, which are differentiated across three age classes but not by income; households can choose different insurance programmes (characterized by higher or lower premiums) depending on the level of deducible and maximum coverage. Premiums are set at the regional level by each health insurer and consequently, they are significantly different across regions and cantons. In addition, households pay directly for healthcare services through out-of-pocket expenditure, deductions (about 330 CHF, in the standard contract), co-payments, and voluntary complementary insurance. As insurance premiums and household expenditure are not dependent on income level, these two forms of financing are regressive. The state, through lower tiers of jurisdictions/local governments, finances a further component of healthcare expenditure by providing subsides to low-income households so that premiums do not exceed 10% of household income, although different eligibility rules are set in different cantons. This contribution is financed through a mixed system using direct taxation (progressive but different across cantons) and indirect taxation through value-added tax (regressive). Depending on the proportion of the two taxes, the overall impact of this source could be regressive, progressive, or even proportional [9, 10]. Lastly, general social insurance provides benefits connected to pensions, disability, and accidents. As the social contribution rate paid by citizens is the same for everyone, regardless of income level, this third financing source is expected to be proportional.

Dental care is covered free of charge if it concerns a serious non-preventable illness of the masticatory system or if it is caused by genetic anomalies. In particular, basic treatments (scaling and root planning, decays treatment, teeth extraction, endodontic treatments, removable prosthesis if less than 20 teeth are in contact between the two arches and all the treatments that are a consequence of genetic problems or diseases such as cancers, granulomas, etc.) are allowed by LAMal, which makes provision for all these basic treatments for indigent people.^1^ ‘Luxury’ or advanced treatments (fixed prosthodontic: crown onlay/overlay, implants, bleaching, micro-abrasions, orthodontic treatments, etc.) are not covered by LAMal and they are fully paid out-of-pocket. In state schools, children’s teeth are checked for free once a year. Although the check-ups are free of charge, if the child requires any treatment for tooth decay, the parents must pay but some local cantonal authorities subsidize the cost of necessary dental treatment with special reductions (up to 80% refund of dental fees) according to the different cantonal laws.

The pricing schema of privately supplied dental treatments differ substantially from that applied to treatments covered by LAMal. In general, tariffs for each treatment are not arbitrarily established or negotiated politically but their amount are based on a cost criterion that takes into account personnel costs, operating expenses, and investment costs. Since 1976, a price list of more than 500 dental services has been published by the Swiss Dental Association and each tariff is determined as the product of two components: the number of points attributable to each treatment (PP) (that depends on the type of the treatment) and the value of each tariff point (VTP) according to the self-estimated skill and expertise of the dentist.^2^ For treatments covered by LAMal the value of each tariff point is set, for simplicity, to 3.1 CHF, whereas the value of each tariff point for privately supplied treatments ranges between 3.1 and 5.8 CHF. Therefore, the pricing scheme applied to privately supplied treatments allows to take into account, on the one hand, the particular circumstances of the patients (urgency, comfort, aesthetics, quality) and, on the other, the particularities of the dental practice (infrastructure costs, wages).^3^ Five minutes of basic dental treatment of a professional dentist correspond to 9 tariff points (PP = 9); therefore, the basic hourly cost of a Swiss dentist ranges from 335 CHF (if VTP = 3.10) to 626 CHF (if VTP = 5.8). Conversely, under the LAMal regime, no adjustments in price are allowed depending on patient’s condition, on dentist’s experience or on materials used and the hourly wage equals 335 CHF (i.e.: VTP = 3.10).

## Data and methods

Different datasets and methods have been used to answer the two research questions posed. Due to the large share of out-of-pocket expenditure in dental care, the vast administrative databases are not helpful in the evaluation of the actual impact of the Swiss reform and there is a substantial paucity of data. Therefore, the main sources of information that we used for this study are three Swiss surveys: the Swiss Household Panel^4^ (year 2014), the Swiss Labour Force Survey (year 2015) and the Swiss Earnings Structure Survey (year 2014). The former dataset has been used to evaluate the presence of barriers to access dental care (1st research question), whereas the last two surveys are employed in the analysis of the economic sustainability of the reform (2nd research question).

The principal aim of the Swiss Household Panel (SHP) is to observe social change, in particular the dynamics of changing living conditions and representations in the population of Switzerland. The survey, run in 16 waves between 1999 and 2014, contains a set of questions concerning several aspects of daily activity and of the financial situation of a subset of Swiss households (7517 households and 18,021 individuals in 2014).^5^ Two of the questions are of interest for our analysis on barriers to access dental care: ‘*Are you or any other member of your household able to go to the dentist if needed?’* and ‘*If no, is it because you cannot afford to do it or for another reason?’*. The analysis of this data allows us to trace the situation within the different Swiss cantons and to highlight the heterogeneity of dental unmet needs across the country. To identify the determinants of reported unmet dental needs, we performed four logistic regressions on the SHP data (year 2014) using different sets of covariates to describe the ‘socio-economic status’ of households. In particular, we run four regressions to decompose the impact of the different covariates on the level of unmet needs for dental care: 1) income alone (Model 1); 2) region of residence alone (Model 2); 3) income and region of residence (Model 3); and 4) a set of socio-economic determinants of the household (i.e. number of members; if it is located in a rural area; its average educational level; labour status of its members; and nationality of its members) plus income and regional dummies (Model 4). Table 1 presents a detailed description of the variables included in the modelling.^6^

**Table 1:** Variables used in the logistic regressions

Variable Name	Description	Note	Models
UNMET NEED	Dependent indicator variable = 1 if the household reported unmet needs for dental care.	The variable assumes value one if the respondent declares that he/she cannot Go to the dentist if needed.	1-4
INCOME	Yearly household equivalised net income (in thousands of CHF). SKOS equivalence scale has been used.	The SKOS scale attributes a weight of 1 to a 1-person household, 1.53 to a two-person household, 1.86 to a three-person household, 2.14 to a four-person household, 2.42 to a five-person household, 2.70 to a six-person household, 2.98 to a seven-person household and increases by 0.28 to each additional person.	1, 3, 4
REGIONAL DUMMIES	7 dummy variables, one for each Swiss region (Lake Geneva; Middleland; North-west Switzerland; Zurich; East Switzerland; Central Switzerland; Ticino).	The dummy variable relative to Lake Geneva has been omitted to avoid collinearity and therefore, Lake Geneva represents the reference region.	2, 3, 4
NC	Number of components in the household.		4
NOTURBAN	Dummy variable = 1 if the household is in a rural or not urban area.	Not urban areas include = Peripheral urban communes; Rural commuter communes, Mixed agricultural communes; Peripheral agricultural communes.	4
EDUCATION	Average number of years of education among family components aged 18 years or more.		4
UNEMPLOYED	Dummy variable = 1 if at least one member of the household is unemployed.		4
FOREIGN	Dummy variable = 1 if at least one member of the household has a foreign nationality.		4

In order to give an economic evaluation of the actual sustainability of the Swiss reform we made use of the data from the other two surveys of the Swiss Statistical Office. The Swiss Labour Force Survey (SLFS) is a telephone survey based on a sample of some 105,000 interviews sent out to individuals every year. The main purpose of the SLFS is to provide information on the structure of the labour force and employment behaviour patterns. The Swiss Earnings Structure Survey (ESS) is a written survey of approximately 35,000 private and public enterprises or administrations with some 1.6 million employees (situation for the 2014 ESS), carried out every two years in enterprises in Switzerland. It allows a regular description to be made of the earnings structure in all economic activities of the secondary and tertiary sectors based on representative data. Using data from SLFS and ESS we obtain and estimate of the 1% wage-based contributions to finance the insurance system. The Swiss Federal Statistical Office provides information on only three quartiles of income distributions: by age class and gender. Using median income as a reference for the computations might lead to serious underestimation of the total 1% contribution. Consequently, we decided to estimate average income by age, class, and gender, estimating for each group a log-normal income distribution and using the corresponding mean as a reference for our computations.

## Results

According to the SHP data, on average, 4.1% of Swiss households reported unmet needs for dental care in 2014, but a preliminary, descriptive, analysis of the data clearly shows that the level of unmet needs is not uniformly distributed across the cantons (Table 2). For example, Ticino and Lake Geneva regions report the highest levels of unmet dental needs (9.3% and 7.8%, respectively), whereas Zurich and Central Switzerland regions record the lowest values (2.7% and 1.7%, respectively). It is undeniable that income plays a role in these percentages; Ticino records the highest percentage of households in the first quartile (lowest income) of Swiss income distribution (39%), followed by Lake Geneva (27%) and East Switzerland (27%). However, income is not the sole factor that can justify the different levels of unmet needs across Switzerland. 20% of households living in Lake Geneva region reported unmet dental needs, while this percentage accounts for only 4% in the cantons of Central Switzerland (Table 2, third column). These disparities, not directly ascribable to income differences, might instead be caused by other socio-economic factors, such as educational level and nationality. If we consider the distribution of unmet needs by nationality, we observe that among households composed by at least one foreigner, 8.11% reported unmet dental needs compared to 2.4% of Swiss households. In addition, people reporting unmet dental needs have, on average, fewer average years of education.

**Table 2:** Percentage of households reporting unmet needs for dental examination or treatment in 2014

Region of residence	% households reporting unmet dental needs	% households in the first quartile of Swiss income distribution	% households reporting unmet needs in the first quartile of regional income distribution	Sample size
Lake Geneva (VD, VS, GE)	7.8	27.1	19.8	1154
Middleland (BE, FR, SO, NE, JU)	3.2	25.3	7.8	1695
North-west Switzerland (BS, BL, AG)	3.5	22.1	10.0	953
Zurich (ZH)	2.7	22.2	8.9	1128
East Switzerland (GL, SH, AR, AI, SG,GR, TG)	3.7	27.4	6.4	857
Central Switzerland (LU, UR, SZ, OW, NW, ZG)	1.7	22.3	4.1	660
Ticino (TI)	9.3	39.3	16.4	253
Total	4.1	25.2	10.6	6700

Source: Swiss Household Panel, year 2014

Notes: data have been weighted to the Swiss population; income refers to yearly household equivalized income (SKOS equivalence scale)

**Fig. 1:**
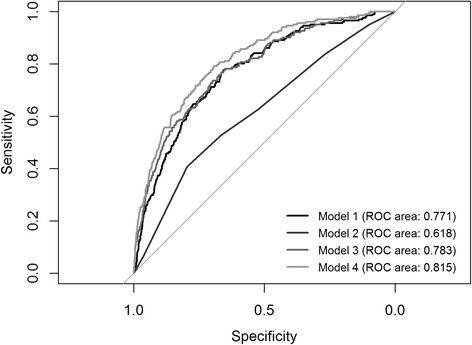
ROC areas in Models 1, 2, 3, and 4

Table 3 gives the results of the four estimated logistic regression models described in Table 1, while a comparison of their goodness of fit using the receiver operating characteristics (ROC) area is given in Fig. 1.

**Table 3:** Results of the logistic regressions in terms of odds ratio

	Model 1	Model 2	Model 3	Model 4
	Odds Ratio	Odds Ratio	Odds Ratio	Odds Ratio
INCOME	0.957***		0.957***	0.963***
	(0.000)		(0.000)	(0.000)
MIDDLELAND		0.385***	0.361***	0.413***
		(0.003)	(0.003)	(0.004)
NORTH-WEST SWITZERLAND		0.428***	0.457***	0.505***
		(0.004)	(0.005)	(0.005)
ZURICH		0.321***	0.347***	0.384***
		(0.003)	(0.003)	(0.005)
EAST SWITZERLAND		0.446***	0.422***	0.484***
		(0.004)	(0.004)	(0.005)
CENTRAL SWITZERLAND		0.204***	0.212***	0.260***
		(0.003)	(0.003)	(0.004)
TICINO		1.199***	0.856***	0.738***
		(0.013)	(0.009)	(0.008)
NC				0.809***
				(0.002)
NOTURBAN				0.726***
				(0.004)
EDUCATION				0.939***
				(0.001)
UNEMPLOYED				1.983***
				(0.023)
FOREIGN				3.134***
				(0.020)
CONSTANT	0.407***	0.085***	0.788***	1.180***
	(0.003)	(0.000)	(0.007)	(0.020)
ROC Area	0.771	0.618	0.783	0.815
Number of observations	6700	6700	6700	6700
Chi^2^ test *p*-value for differences in ROC areas	1.3e + 05 ***			

Notes: *** = *p*-value < 0.001. Standard errors are in brackets

Model 1 (ROC area = 0.77) shows that the higher income is, the lower is the probability of experiencing unmet needs. The six regional dummies in Model 2 (ROC area = 0.62) turn out to be significant, indicating that, compared to the Lake Geneva reference region, the probability of reporting unmet needs for dental care increases for those families living in the region of Ticino but decreases for all other regions. Model 3 (ROC area = 0.78), which combines income and regional dummies, brings an improvement in the goodness of fit compared to Model 1. The addition of other socio-economic drivers in Model 4 (ROC area = 0.82) further improves the explanatory capacity of the model. In particular, the probability of reporting unmet dental needs increases in households in which at least one member is unemployed or of foreign nationality; therefore, unemployment and foreign nationality might be considered risk factors, in accordance with the international literature. In addition, having a higher educational level impacts negatively on the probability of reporting unmet dental needs. Living in rural areas negatively affects the level of unmet needs; this factor is in contrast to previous research [39].

A crucial aspect in evaluating a reform is its economic sustainability, that is, if the resources to be made available by the reform are adequate to cover the new expenses. Table 4 reports the 2014 Swiss workforce in thousands of units and the estimated average gross monthly salary for October 2015 by age and gender. Using average income by age class and gender, estimating for each group a log-normal income distribution and using the corresponding mean as a reference for our computations, we compute the expected contributions derivable from the 1% withdrawal. We obtain an estimate of 3.5 billion CHF, that is 600 million CHF less than the dental care expenses reported by the Swiss Federal Office of Statistics in 2014. This is equivalent to 430 CHF per capita per year (85% of actual dental care expenditure). Since basic dental treatment is already guaranteed to all individuals, a reform of the current system could be justified only if the financing plan for the new insurance scheme would significantly improve the basket of guaranteed dental services; however, given the estimations, improving the basket of guaranteed dental services does not seem possible through the suggested financing scheme.

**Table 4:** Gross monthly wage and workforce by age and gender and estimated contributions

Age	Workforce (× 1000)			Estimated average monthly wage			1% contribution per year (× 1000)		
	Men	Women	Total	Men	Women	Total	Men	Women	Total
15–24	282	282	564	4761	4518	4640	161,110	152,893	314,004
25–39	782	684	1466	6091	5594	5859	571,542	459,154	1,030,695
40–54	887	774	1661	7418	6310	6902	789,612	586,060	1,375,672
55–64	399	338	737	8113	6403	7329	388,442	259,715	648,158
65+	105	67	172	9731	6473	8462	122,613	52,043	174,656
Total	2455	2145	4600	6902	5866	6419	2,033,320	1,509,865	3,543,185

Sources: Gross monthly wage is from the Swiss Federal Statistical Office, Enquête Suisse sur la population active [16] and workforce by age and gender is from the Swiss Federal Statistical Office [42]

## Discussion

According to the Swiss Federal Statistical Office, in 2014, households directly covered roughly 68% of the total cost of healthcare financing (through premiums and direct household expenditure) and 90% of dentists’ cost (through out-of-pocket expenditure). As stated earlier, the largest part of the total cost of healthcare and an even the largest part of dental care expenditure is financed through a regressive type of contribution. The introduction of a contribution for dental care proportional to income (and half covered by employers) is likely to change the financial burden borne by low-income households; in the post-reform financing scheme, a new proportional contribution would indeed replace a regressive source of financing, decreasing the regressivity level of the whole system. Even if the available datasets are not sufficient to assess what kind of dental services are most demanded depending on the income level, it should be considered that most international literature is concordant in assessing that, even when freely provided, dental healthcare services (particularly the specialist ones) are more intensively used/demanded by people with a higher than average level of income. Consequently, it cannot be taken for granted that the reform would favour the most vulnerable individuals, who tend in any case to use less dental services, irrespective of dental care price. Second, Switzerland already has set up an effective system of protection of vulnerable individuals. According to LAMal, insurance premiums indeed should be reduced for people of modest economic circumstances by federal and cantonal contributions. The amount and nature of premium reduction and the entitlement conditions, however, are set by single cantons and therefore, vary widely. Overall, French-speaking cantons tend to be more generous than German-speaking ones. Therefore, a set of dental services (emergency treatment and sanitation, conservative treatment, and prosthetic restoration) is already guaranteed to low-income individuals. For example, in the Canton of Geneva, the *Service des Prestations Complémentaires* already covers emergency care and all periodontal treatments independently of the severity of the clinical case. All basic operative treatments are guaranteed, such as endodontic treatments and direct composites. Even missing teeth are replaced by means of removable prosthesis. On the other hand, all ‘luxury treatments’, such as orthodontic treatments to re-align teeth, bleaching, implants, and fixed crowns, are not covered by this social insurance.

The relevance of geographical factors and socio-demographic characteristics of households in explaining unmet needs is particularly interesting, as it suggests that, given fixed income, the socio-economic status and cultural factors intrinsic to each region might affect the perceived need for dental services. As the presence of barriers to access dental services is a crucial point raised by the proponents of the reform, it is necessary to pay attention to basing similar reform on considerations of the level of unmet needs alone. Income is not a unique driver of barriers to access dental care. The relevance of nationality and educational level in explaining self-reported needs might be interpreted as a need for more educational and informative campaigns addressed to foreign and low-educated individuals. Indeed, preventive programmes emphasising care for the erupting molars, the use of a firm guideline and stated goals to be achieved showed to be extremely effective whatever the patients’ socio-economic group (e.g.: Ekstrand and Christiansen [15] on the non-operative caries treatment programme (NOCTP) used since 1987 in the municipality of Nexö in Denmark). In addition, the relevance of geographical dummies suggests that measuring barriers to access through a self-reported measure might suffer from perception/attitude bias intrinsic to each region (i.e. in this respect, Swiss Germans are apparently different from Swiss French). In particular, useful policies could include informative and preventive campaigns aimed at reducing the current informative gap between foreign- and native-born individuals and between high- and low-dental hygiene concerned individuals.

The possible effects of the reform might be influenced by several phenomena whose impacts are hardly predictable. First, the aforementioned dental expenses of 4.1 billion CHF include both basic treatments (periodic controls, dental hygienist, prevention, and conservative caries treatment) and advanced treatments (bridges and crowns), but it cannot be ignored that there is potential dental care demand that is unmet because of household budget constraints. This sustainability analysis risks being optimistic as it completely ignores the likely increase in patients’ way of using dental care once it is covered by compulsory insurance [28]. Estimating the change in demand from the introduction of compulsory insurance is a challenging task due to the paucity of similar international experiences. According to SHP data 4.1% of Swiss people without any dental insurance reported unmet needs for dental examinations and treatments during 2014. It is reasonable to believe that a large share of people reporting unmet needs for economic reasons will instead be able to satisfy their demand under a compulsory insurance scheme. If we assume an additional cost of 41 CHF a month for 4.1% of Swiss people (i.e. those reporting unmet needs), this would lead to a further increase in costs equal to roughly 139 million CHF per year. Having expenses covered by insurance, it is likely that the demand for advanced (and costly) treatments would increase further but there are no data that might help in defining/quantifying the amount of such an increase.^7^

Finally, the health insurance loading fee, that is, the portion of the premium that insurance companies use to cover general and claims-related expenses, should be taken into account. According to the Swiss Federal Office of Public Health [6], in 2014, the premiums due for compulsory health insurance were 25,845 million CHF while the administrative costs were 1287 million CHF, with the share of expenditure ranging from a minimum of 2.9% to a maximum of 14.3%, for an average 4.9%. A conservative scenario is to extend the 5% average health insurance loading fee to dental insurance: starting from the 3.5 billion CHF estimation we obtained earlier, we found that the administrative fees would be around 175 million CHF.

## Conclusions

Our findings highlight the issues currently at stake in the evaluation of the eventual reform of the Swiss dental care system. First, income is possibly the main driver of unmet needs for dental care but not the unique. The association between a worse dental status and lower sociodemographic level is the effect a complex interaction of various risk factors. In particular, periodontal diseases and dental decay are mainly originated from bad oral hygiene, lack of basic cleaning procedures, and irresponsible diet and habits (e.g. excess of sugar-based food and smoking) that are more common in low level sociodemographic groups, the ones that experience economic barriers to dental care access. However, Schneider et al. [38] outline that in Switzerland in the period 1992-2012 “dental status improved across all sociodemographic groups, with the greatest improvements being found in obese participants and in participants with the lowest incomes and educational levels” and that “even though dental treatment continues to be a self-paying system, the differences between sociodemographic groups decreased markedly”. From the non-economic barriers side, dental status disparity among the different sociodemographic groups could be substantially reduced funding preventive policies addressed to foreign individuals and low-educated people breaking-down these barriers to dental care access. Indeed, international literature shows that preventive dental care sessions, and NOCTP in particular [15], substantially improve the dental health status of a community. On the other side, although the proponents claim that the reform represents the solution to the economic barriers to dental care access, it worth recalling that LAMal already provides a structured protection system for vulnerable individuals covering several dental services with the exception of luxury dental treatments. Moreover, considering that insurance coverage may lead to an increase in demand for dental services, if the 1% contribution is the only way that proponents of the reform suggest covering dental care costs (and by taking into consideration the fact that the administrative expenditure will reduce the collected total amount by around 5%), the remaining annual per capita amount of 409 CHF seems to be inadequate to bring an improvement in public dental health. On the supply side, dentists associated with insurance companies might have to decrease the value of the VTP to 3.10 CHF (the ‘social tariff’ level in Switzerland) with a possible decrease in quality of the materials and equipment used and of the treatment procedures. In particular, for those cases in which the offices are located in high-priced areas, such as Zurich or Geneva, this fact could lead to serious problems in financing these offices.

Therefore, the proposal for a 1% wage contribution to finance dental care insurance seems inadequate for the stated purpose (i.e. to guarantee all ‘free’ access to advanced dental care treatments, e.g. teeth replacements by means of implants, bridges, and orthodontic treatments). With the expected revenue, it would be possible to provide the entire population with only basic preventive 1-h sessions with dentists for simple basic preventive measures, which is nowhere near being sufficiently efficient to avoid the development of periodontal or caries symptoms, such as periodontal pockets or carious lesions. Apart from this, it has to be pointed out that this kind of service could be provided in Switzerland by dental hygienists, whose hourly prices are around 60% cheaper than those of dentists (3.5 PP vs 9 PP for a 5 min session). A prophylaxis assistant costs roughly 65,000 CHF per year, and has capacity to work around 1800 h per year. In addition, expanding the pool of people with access to social insurance programmes might be a viable and cheap way to guarantee preventive and prophylaxis treatments to more adults. The additional resources for this counter-proposal could be financed by a tax on sugary drinks (soft drinks, fruit juices, and energy drinks) as well as a small increase in taxes on tobacco. Denmark has adopted best practice to support children and adolescents in this sense [4, 15, 17, 21, 43]. The costs of this action would be significantly lower than the dental insurance initiative, because the former system specifically targets the at-risk group of patients and primarily works with prophylaxis assistants.

To conclude, given the available information, the expectation of ‘free’ dental care for all seems to be hardly achievable, except if the significant missing funds were guaranteed by cross-financing through increases in general communal, cantonal, or federal taxes. Consequently, reinforcement of prevention aimed at preventing dental illness is preferable to treatment of the illness itself.

## Abbreviations

CHF: Swiss Francs
ESS: Swiss Earnings Structure Survey
LAMal: Loi fédérale sur l’assurance-maladie
MHI: Mandatory Health Insurance
OECD: Organisation for Economic Co-operation and Development
ROC: Receiver Operating Characteristics
SHARE: Survey of Health, Ageing and Retirement in Europe
SHP: Swiss Household Panel (SHP)
SLFS: Swiss Labour Force Survey
SSO: Société Suisse d’Odonto-Stomatologie

## Footnotes

1 The criteria for determining a person “indigent” are income and the number of children and differ among the 26 Swiss Cantons.

2 Dental tariffs can be downloaded from: https://www.sso.ch/fr/patients/droit-et-tarif/tarif-dentaire.html

3 During 2018 both tariff points (PP) and values (VTP) will be rescaled so that each point will cost between 1 and 1.6 CHFs according to the dentist’s skill and expertise.

4 The Swiss Household Panel (SHP) is based at the Swiss Centre of Expertise in the Social Sciences FORS, financed by the Swiss National Science Foundation.

5 In the analyses, microdata have been weighted to be representative of the Swiss population.

6 Due to missing data on income, the total number of observations used in regression models drops to 6700.

7 Due to the lack of survey or administrative data on the demand and expenses for basic and advanced dental treatments, it is not possible to know what proportion of patients used advanced dental care services. Consequently, the estimations herein proposed are based on simple averages and should be read with caution.
